# Exploring the role of galectin-9 and artemin as biomarkers in long COVID with chronic fatigue syndrome: links to inflammation and cognitive function

**DOI:** 10.3389/fimmu.2024.1443363

**Published:** 2024-09-25

**Authors:** Shokrollah Elahi, Maryam Rezaeifar, Mohammed Osman, Shima Shahbaz

**Affiliations:** ^1^ School of Dentistry, Division of Foundational Sciences, Edmonton, AB, Canada; ^2^ Li Ka Shing Institute of Virology, Edmonton, AB, Canada; ^3^ Women and Children Health Research Institute, Edmonton, AB, Canada; ^4^ Cancer Research Institute of Northern Alberta, Edmonton, AB, Canada; ^5^ Glycomics Institute of Alberta, Edmonton, AB, Canada; ^6^ Alberta Transplant Institute, Edmonton, AB, Canada; ^7^ Department of Medicine, Division of Rheumatology, Faculty of Medicine and Dentistry, University of Alberta, Edmonton, AB, Canada

**Keywords:** long COVID, chronic fatigue syndrome, galectin-9, artemin, microbial translocation, HIV

## Abstract

This study aimed to assess plasma galectin-9 (Gal-9) and artemin (ARTN) concentrations as potential biomarkers to differentiate individuals with Long COVID (LC) patients with myalgic encephalomyelitis/chronic fatigue syndrome (ME/CFS) from SARS-CoV-2 recovered (R) and healthy controls (HCs). Receiver operating characteristic (ROC) curve analysis determined a cut-off value of plasma Gal-9 and ARTN to differentiate LC patients from the R group and HCs in two independent cohorts. Positive correlations were observed between elevated plasma Gal-9 levels and inflammatory markers (e.g. SAA and IP-10), as well as sCD14 and I-FABP in LC patients. Gal-9 also exhibited a positive correlation with cognitive failure scores, suggesting its potential role in cognitive impairment in LC patients with ME/CFS. This study highlights plasma Gal-9 and/or ARTN as sensitive screening biomarkers for discriminating LC patients from controls. Notably, the elevation of LPS-binding protein in LC patients, as has been observed in HIV infected individuals, suggests microbial translocation. However, despite elevated Gal-9, we found a significant decline in ARTN levels in the plasma of people living with HIV (PLWH). Our study provides a novel and important role for Gal-9/ARTN in LC pathogenesis.

## Introduction

Long COVID (LC) is a major global health concern that has impacted the quality of life of millions of individuals. It is a multisystemic condition with incidence rates ranging from 10 to 70% depending on the studied cohorts and the time of screening (1–3). LC can be observed in any age group, but it appears to be more common between the ages of 30 and 50 years. Additionally, it is frequent to observe more LC cases in those with mild acute COVID-19 disease (1, 4). This suggests that LC can occur regardless of the severity of acute disease and in the absence of other co-morbidities. Notably, women are disproportionality impacted, with more severe symptoms associated with LC and more frequently than men (4, 5).

While LC improves over time in some patients, others continue to experience LC symptoms even for years. Unfortunately, a subset of patients exhibits the most debilitating form of LC, myalgic encephalomyelitis/chronic fatigue syndrome (ME/CFS), and this might be lifelong in a subset of them (1, 6). LC patients experience a wide range of symptoms, such as cardiovascular and thrombotic diseases, cerebrovascular disease, ME/CFS, dysautonomia, autoimmune conditions, cognitive impairment, etc.

Nevertheless, the molecular mechanism underlying the most post-acute disease symptoms, including ME/CFS remains not fully understood. Several potential mechanisms have been proposed, including metabolomic and immune dysregulation, chronic inflammation, dysregulated hematopoiesis, hypoxia, viral persistence, autoimmunity, alteration of microbiota and gastrointestinal inflammation, endothelial dysfunction, and impaired signaling in the brainstem and/or vagus nerve (1, 4, 7–12). Although fatigue is one of the main symptoms in this group, the major symptom observed in a subset of LC patients is cognitive impairment, or brain fog, which has been linked to autonomic nervous system dysfunction (13). Given the blood-brain barrier (BBB) disruption, neurological symptoms (e.g. brain fog) observed in LC patients are likely due to sustained systemic inflammation and BBB dysfunction (14). Indeed, a peripheral immune-mediated response to viral antigens and neuroinflammation might indirectly result in neurocognitive symptoms (15). Mechanistically, it is known that in some cases circulating pathogen associated patterns (PAMPs) can reach the CNS and choroid plexus (CP) and the circumventricular reigns (16). For example, peripheral administration of LPS and/or inflammatory cytokines are associated with impaired learning skills in animal models (17, 18). Therefore, it is possible to speculate that an unresolved localized or systemic pro-inflammatory state contributes to CNS symptoms in LC patients. In this scenario, residential cells in the CNS are exposed to systemically produced pro-inflammatory cytokines, chemokines, and other damage-associated molecules (DAMPs), ultimately resulting in neurological symptoms, fatigue, and neurocognitive impairment in a subset of LC patients.

Galectin-9 (Gal-9) is a β-galactosidase binding lectin with diverse immunomodulatory properties (19). It is widely abundant in immune and non-immune cells and binds to different receptors, such as TIM-3, PD-1, PDI, IgE, CD44, CD45, and CD3 among others (20–24). The concentration of soluble Gal-9 is reported to be elevated in the plasma, saliva, and synovial fluids in HIV and other inflammatory conditions (25–27). Gal-9, as a DAMP, has been reported to play a predominant role in cytokine release storm in the acute SARS-CoV-2 infection (28). Most recently, we reported elevated levels of Gal-9 in the plasma of LC patients with ME/CFS (29). In particular, our findings supported a positive correlation between the plasma Gal-9 levels with CRP, MIP-1β, IL-10, and VCAM-1 in these patients (29). Of note, persistent gastrointestinal (GI) symptoms are reported in acute phase of COVID-19 disease and LC patients (30, 31). This may, in part, suggest the presence of compromised GI mucosal integrity in LC patients. Thus, plasma soluble CD14 (sCD14) and intestinal fatty acid binding protein (I-FABP) are considered reliable biomarkers for assessing GI permeability and epithelial integrity (32, 33). However, the association of these biomarkers with Gal-9 has not been documented in LC. Moreover, elevated Gal-9 expression across brain tissues is reported to be associated with neuropathology and cognitive impairment in HIV-infected individuals (34).

Additionally, we have reported the elevation of artemin (ARTN), a neurotrophic factor, in the plasma of LC patients with ME/CFS (29). ARTN interacts with its receptor GFRα3 and its co-receptor RET affecting cell growth and differentiation (35). Notably, the role of ARTN in neuropathic pain has been the subject of debate (35, 36). We previously reported that systemic ARTN levels were positively correlated with the cognitive impairment and pain scores in LC patients (29). Therefore, in the current study, we decided to determine whether plasma Gal-9 levels have any association with cognitive impairment scores in LC patients with ME/CFS, as reported in people living with HIV (PLWH) (34, 37). Given that compromised gut integrity and microbial translocation are contributing factors to chronic inflammation commonly observed in PLWH (32, 38), due to this similarity, we decided to examine whether this is the case in LC patients by measuring LPS-binding protein (LPS-BP). Finally, considering the association of plasma ARTN levels with cognitive impairment scores in LC patients with ME/CFS (29) and reported HIV-associated neurocognitive disorder (34), we quantified this neurotrophic factor in the plasma of PLWH and compared them with values of the other groups. Taken together, our findings suggest that plasma Gal-9 and ARTN levels, with high sensitivity and specificity, can differentiate LC from the recovered group. Furthermore, we observed a positive correlation between plasma Gal-9 with inflammatory biomarkers such as sCD14, I-FABP, LPS-BP, serum amyloid A (SAA), and IP-10 in LC patients. Notably, we found that Gal-9 was positively correlated with cognitive failure score in LC patients as reported in PLWH (37, 39). Despite the reported elevation of Gal-9 in the plasma of PLWH, these individuals had significantly lower levels of ARTN compared to healthy individuals and LC patients.

## Methods

### LC cohort

The first cohort (discovery) comprised 44 LC patients (median age 51.5±13.1, 11 males and 33 females) and 24 SARS-CoV-2 infected individuals who had recovered (R) from the disease without any obvious symptoms and complications (median age 50.5 ± 13.3, 6 males and 18 females). All were infected with the original Wuhan SARS-CoV-2 strain. All study subjects were recruited approximately 12 months (371±19 days LC vs. 368±6.2 days R) after the onset of SARS-CoV-2 infection as reported elsewhere (29). We utilized a set of well-defined validated clinical questionnaires developed by CDC and WHO (40, 41) for the diagnostic of LC patients with ME/CFS. To determine the applicability of our findings to another cohort, we established a validating cohort. The validating cohort consisted of 34 LC patients (median age 48 ± 9.8, 9 males and 25 females) and 34 recovered individuals (median age 45 ± 11.39, 10 males and 24 females) from SARS-CoV-2 infection without any symptoms. The infection was confirmed by PCR in both cohorts. This validating cohort was infected mainly with the Delta/or Omicron variants. Similar to the discovery cohort, they were recruited approximately 12 months (435±89 days LC vs. 415±40 days R) after the onset of acute disease, as reported elsewhere (29).

Study participants were age-and sex-matched, and considering that the majority of our patients had a mild acute infection, confounding health conditions were not common. All study subjects (LC and R) in the first cohort were SAS-CoV-2 vaccine-naïve but 67.3% of LC and 73.5% of R were vaccinated in the validating cohort. All of our LC patients in both cohorts met the criteria for categories I, II, III, IV, V, and VI associated with ME/CFS as we previously reported (29). Those LC patients who did not meet the criteria established by CDC and WHO (40, 41) were excluded from the study. Considering that a subset of PLWH presents cognitive impairments, we decided to compare our findings with a cohort of PLWH.

### HIV cohort

Our HIV group comprised 63 individuals on antiretroviral therapy (ART) form the Northern Alberta HIV cohort as reported elsewhere (24, 42). We also recruited 25 healthy controls who were serologically negative for HIV, hepatitis C virus (HCV), and hepatitis B virus (HB) for comparison.

### Ethics statement

The COVID-19-related study was approved by the Human Research Ethics Board (HREB) at the University of Alberta (protocol # Pro00099502). Similarly, the HIV-related study was approved by the HREB (protocol # Pro00070528) and HCs (protocol # Pro00063463). A written informed consent form was obtained from all participants.

### Cytokine and chemokine multiplex analysis and ELISA assays

Frozen plasma samples stored at -20/80°C were thawed and centrifuged for 15 min at 1500g followed by dilution in PBS for quantifying cytokine/chemokine and other soluble analytes. The concentration of cytokines and chemokines was quantified using the V-PLEX Neuroinflammation panel 1 kit (K15210D-1) from Meso Scale Discovery (MSD) (28, 43). Additionally, the plasma was subjected to ELISA assays for Gal-9 (R&D, DY 2045), ARTN (R&D, DY 2589), LPS-Binding protein (DY870-05), FABP-1 (R&D, Z-001), and sCD14 (DC140), as we have reported elsewhere (21, 28, 43).

### Statistical analysis

Spearman correlation was used to measure association between two variables. The non-parametric Mann-Whitney U test for two groups or One-way ANNOVA was used when more than two groups were compared. Measures are expressed as mean ± SEM, and a *P-*value < 0.05 was considered to be statistically significant.

## Results

**Figure 1:**
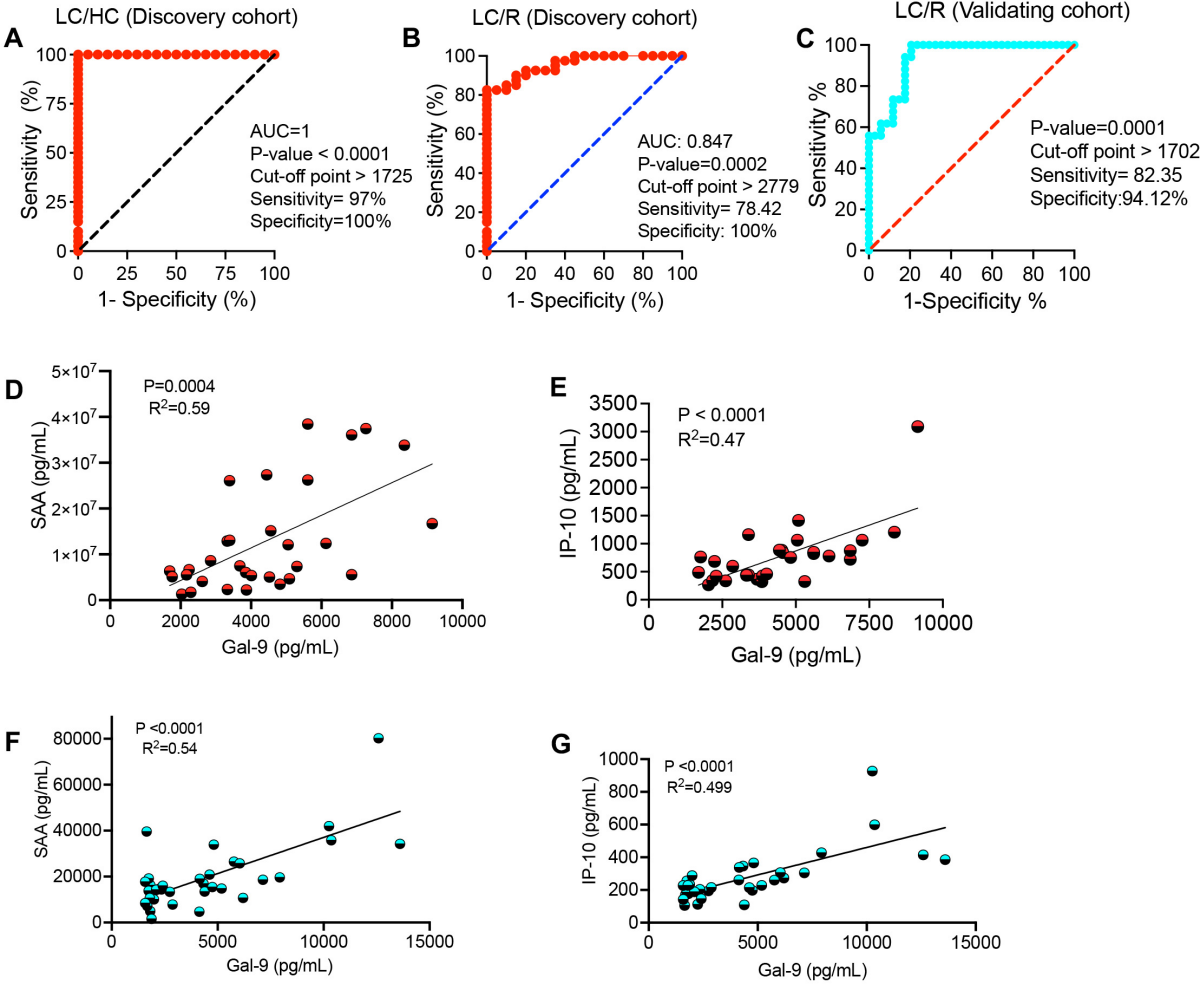
The diagnostic value of galectin-9 (Gal-9) in Long-COVID patients. **(A)** The receiver operating characteristic (ROC) curve for Gal-9 in LC versus healthy controls (HC) in the discovery cohort. **(B)** The ROC curve for Gal-9 in LC versus recovered individuals (R) in the discovery cohort. **(C)** The ROC curve for Gal-9 in LC versus R in the validating cohort. **(D)** The correlation between plasma Gal-9 and SAA, and **(E)** IP-10 levels in LC patients of the discovery cohort. **(F)** The correlation between plasma Gal-9 and SAA, and **(G)** IP-10 levels in LC patients of the validating cohort.

Recently, we reported elevated levels of Gal-9 and ARTN in the plasma of LC patients with ME/CFS (29). To further investigate whether plasma Gal-9 and/or ARTN concentrations could serve as surrogate biomarkers to differentiate LC patients from recovered (R) and/or HCs, we conducted additional analysis. Using the receiver operating characteristic (ROC) curve, we calculate the optimal cut-off value of Gal-9 to distinguish LC patients from HCs. The ROC curve point with the best sensitivity/specificity indicated that a plasma Gal-9 level greater that 1725 pg/ml separates LC from HCs with 97% sensitivity and 100% specificity (**Figure 1A**). To identify LC patients from the R group, we determined a Gal-9 cut-off value of greater than 2779 pg/ml, achieving a sensitivity of 78.5% and specificity of 100% (**Figure 1B**). This finding was confirmed in our validating cohort, where a cut-off value of greater than 1702 pg/ml/ml differentiated LC patients from the R group with 82.35% sensitivity and 95% specificity (**Figure 1C**). These observations suggest that the plasma Gal-9 is a sensitive screening biomarker for discriminating LC patients with ME/CFS from both R and HCs.

In addition to our previous findings of a positive correlation between plasma Gal-9 and CRP, VCAM-1, MIP-1β, and IL-10 (28, 44), we observed similar correlations between plasma Gal-9 and SAA and IP-10 levels in our discovery (**Figures 1D, E**) and validating LC cohorts, respectively (**Figures 1F, G**).

**Figure 2:**
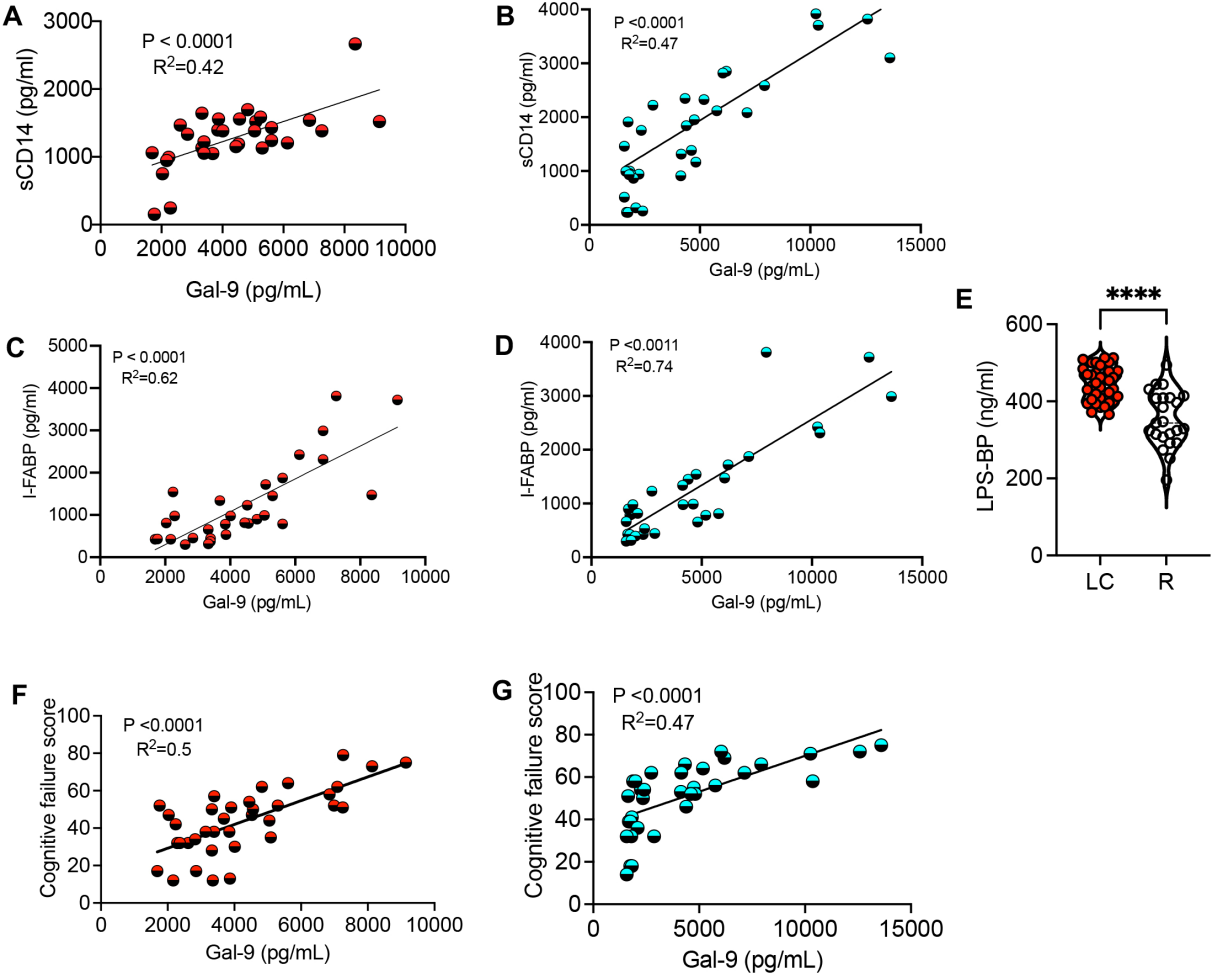
The correlation of Gal-9 with inflammatory and cognitive impairment in LC patients. **(A)** The correlation between plasma sCD14 and Gal-9 in the discovery, and **(B)** validating cohort. **(C)** The correlation between plasma I-FABP and Gal-9 in the discovery, and **(D)** validating cohort. **(E)** Detected concentrations of LPS-binding protein (LPS-BP) in the plasma of LC versus R in both cohorts. **(F)** The correlation between plasma Gal-9 and cognitive failure score in the discovery, and **(G)** validating cohort. The symbol **** shows a p value less than 0.0001 or P < 0.0001.

**Figure 3:**
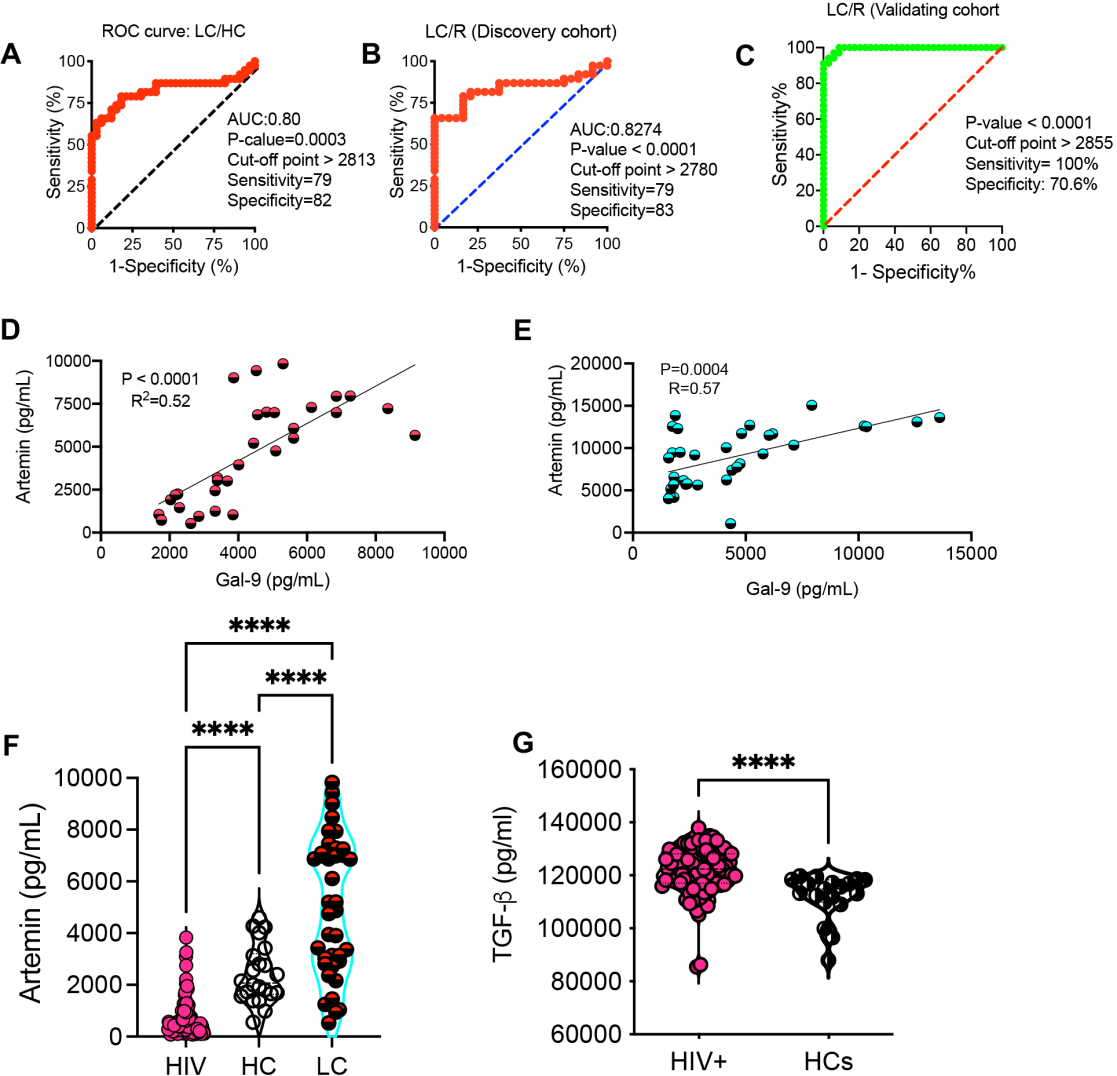
The diagnostic value of artemin (ARTN) in Long-COVID patients. **(A)** The ROC curve for ARTN in LC versus HCs in the discovery cohort. **(B)** The ROC curve for ARTN in LC versus the R group in the discovery cohort. **(C)** The ROC curve for ARTN in LC versus R in the validating cohort. **(D)** The correlation between plasma Gal-9 and ARTN in LC patients of the discovery, and **(E)** validating cohort. **(F)** Detected ARTN levels in plasma samples from HIV-infected individuals, HCs and LC patients of both cohorts. **(G)** TGF-β levels in plasma samples from HIV-infected individual versus HCs. The symbol **** shows a p value less than 0.0001 or P < 0.0001.

Activated innate immune cells (e.g. neutrophils and monocytes) can shed Gal-9 (20, 28). Given the elevated sCD14 in the acute phase of COVID-19 disease (44) and in LC patients (29), our observations suggest that Gal-9 may contribute to the elevation of sCD14 in the plasma of LC patients. As such, we found a moderate positive correlation between plasma Gal-9 and sCD14 concentrations in LC patients across both discovery and validating cohorts (**Figures 2A, B**). Furthermore, with the elevation of the I-FABP in LC patients’ plasma (29), we observed a positive correlation between I-FABP and Gal-9 levels in both cohorts (**Figures 2C, D**). Measurement of LPS-binding protein levels revealed a significant increase in LC patients compared to the R group (**Figure 2E**). Given the association of Gal-9 with HIV neuropathology and cognitive deficits (34, 37, 45), we explored whether plasma Gal-9 is linked to cognitive impairments in LC patients. Our analyzes showed a positive correlation between Gal-9 levels and impaired cognitive function scores in both LC cohorts with ME/CFS (**Figures 2F, G**). However, we did not find any association between Gal-9 levels and other symptoms such as pain severity or widespread pain scores in LC patients (data not shown). Considering the elevated plasma ARTN levels in LC patients (29), we evaluated the diagnostic value of ARTN concentrations as a non-invasive biomarker in these patients. A cut-off value of greater than 2813 pg/ml plasma ARTN distinguished LC from HCs with 79% sensitivity and 82% specificity in our discovery cohort (**Figure 3A**). Similarly, a cut-off value of greater than 2780 pg/ml plasma ARTN differentiated LC from R with 79% sensitivity and 83% specificity in the discovery cohort (**Figure 3B**). Notably, in the validating cohort, a cut-off value of greater than 2855 pg/ml ARTN reliably differentiated LC patients from the R group with 100% sensitivity and 70% specificity (**Figure 3C**). The diagnostic values of both Gal-9 and ARTN were supported by a moderate and positive correlation between their plasma concentrations in both LC cohorts (**Figures 3D, E**).

Given the elevated plasma Gal-9 levels in PLWH (21, 24), we examined whether ARTN levels were also increased in HIV infection. Surprisingly, we found a significant reduction in ARTN levels in PLWH compared to HCs (**Figure 3F**). Although ARTN is a member of the TGF-β superfamily (46), its plasma levels were sharply elevated in PLWH compared to HCs (**Figure 3G**). In contrast, TGF-β levels were significantly reduced in LC patients (29). Despite the commonality of chronic immune activation and immune dysregulation in both HIV and LC subjects, ARTN levels were substantially lower in PLWH compared to LC patients (**Figure 3F**).

## Discussion

This study aimed to explore the potential of plasma Gal-9 and ARTN levels as surrogate biomarkers for distinguishing LC patients from HCs and R individuals. The ROC curve analysis revealed that the plasma Gal-9 and ARTN serve as effective biomarkers with high sensitivity and specificity in differentiating LC patients from Rs and HCs. This suggests that Gal-9 and ARTN hold promise as non-invasive screening biomarkers for identifying LC individuals experiencing ME/CFS. Whether these biomarkers are associated with idiopathic ME/CFS requires further investigation.

The reproducibility of our findings in a separate cohort strengthens the evidence that plasma Gal-9/ARTN concentrations could be valuable tools in distinguishing LC patients with ME/CFS.

The observed positive correlations between plasma Gal-9 levels and inflammatory markers such as SAA and IP-10 are consistent with our previous observations during acute SARS-CoV-2 infection and in LC patients (28, 29, 44). This reaffirms the role of Gal-9 as a potential indicator of immune dysregulation in both acute and prolonged phases of COVID-19 disease. However, elevated plasma Gal-9 levels have been reported in various pathological conditions such as HIV infection, virus-associated solid tumors, chronic lymphocytic leukemia, hepatitis C infection, autoimmune hepatitis, influenza infection, and other inflammatory conditions (21, 22, 25, 26, 47–49). Therefore, it is important to consider that other chronic inflammatory conditions may also influence Gal-9 levels in plasma when evaluating this lectin in LC study subjects. Furthermore, it is important to note that Gal-9 interacts with various receptors such as TIM-3, CD45, CD44, CD3, PDI, and PD-1. The biological consequences of these interactions vary significantly depending on the target cell, the expression level of the corresponding receptor, and the microenvironment. For instance, interactions of Gal-9:TIM-3 and Gal-9:PD-1 are associated with CD8+ T cell exhaustion (22, 50, 51). In contrast, Gal-9:CD3 interaction enhances TCR signaling in T cells (20, 52). Similarly, while Gal-9:CD44 interaction promotes NK cell effector functions under physiological conditions, it impairs their cytotoxic capabilities in chronic conditions (23, 53).

Our investigation into potential sources of elevated Gal-9 in LC patients revealed a positive and moderate correlation with plasma sCD14 levels. This association with sCD14 implies that activated innate immune cells (e.g. neutrophils and monocytes) likely shedding this lectin (20, 28) may contribute to the elevated Gal-9 levels in LC patients. In agreement, blocking CD14 has resulted in a substantial reduction in neutrophil abundance in the lung and peripheral blood and subsequently reduced organ dysfunction due to inflammation in COVID-19 patients (54). Given the importance of plasma concentrations of sCD14 in conjunction with I-FABP in compromised intestinal permeability (33, 55), we found a substantial correlation between Gal-9 with sCD14 and I-FABP in LC patients. Moreover, the elevation of LPS-binding protein in the plasma of LC patients further supports the complex interplay between immune activation, compromised GI integrity, and metabolic pathways in LC. The elevation of LPS in the plasma of PLWH is considered as a marker of microbial translocation associated with increased sCD14 and chronic immune activation (56). These observations suggest that compromised intestinal barrier integrity during the early phase of infection or persistent SARS-CoV-2 replication in the GI tract contributes to sustained immune activation and dysregulation in LC patients. This hypothesis is further supported by shedding of fecal SARS-CoV-2 RNA in patients up to 7 months post-acute disease (57).

Therefore, persistent residual viral replication in LC patients, similar to HIV, or the presence of SARS-CoV-2 viral antigens in tissues (e.g. gut) (12), can result in chronic immune activation. Consequently, the pro-inflammatory response and metabolomic alterations (4, 29) may promote the Warburg effect in LC patients. We suggest that the chronic inflammatory state may promote mitochondrial dysfunction in LC patients. The observed reduction in ATP plasma levels in LC patients (4) suggests the potential effects of persistent viral replication/innate immune activation on the glycolytic and mitochondria pathway in LC. It is noteworthy that the Warburg effect may subsequently downregulate T and B cell effector functions, as reported in cancer patients (58). For example, enhanced tryptophan metabolism and increased kynurenine metabolites can impair B cell effector functions (59) and also contribute to musculoskeletal symptoms observed in LC patients (60). Additionally, elevated Gal-9 may enhance SARS-CoV-2 entry in a glycan-dependent manner, as reported via enhanced binding of the spike protein with ACE2 (61). Likewise, enhanced SARS-CoV-2 infection by Gal-3, Gal-8, and Gal-9 has been reported (44). However, the effect of Gal-9 on HIV infection is receptor-dependent. For instance, Gal-9, via interaction with TIM-3, reduces HIV infection, and via interaction with PDI enhances HIV infection in CD4+ T cells (21, 62). Notably, our study unveils a potential link between Gal-9 levels and cognitive function in LC patients as documented in PLWH (37, 39). The positive correlation with cognitive failure scores suggests that Gal-9 may contribute to cognitive impairment in individuals with LC. Whether this effect is indirect through the elevation of pro-inflammatory cytokines/chemokines or direct needs to be determined. It has been reported that Gal-9 acts as an astrocyte-microglia signaling molecule, enhancing cytokine production (e.g. IL-6 and TNF-α) (63). Moreover, elevated levels of plasma and cerebrospinal fluids (CSF) Gal-9 are correlated with cognitive impairments in PLWH (64), further supporting its potential role in cognitive deficits in other viral infections, such as LC. Of note, the potential impact of ART on Gal-9 and other inflammatory biomarkers in PLWH should be considered. Therefore, further studies beyond HIV and LC are needed to evaluate the potential impact of elevated plasma Gal-9 on cognitive functions in other pathological conditions. Nevertheless, the source of Gal-9 needs to be determined, as immune and non-immune cells in the periphery and CNS can express and secrete this lectin (20, 28, 63). Although Gal-9 could originate from the periphery, diffusion between the plasma and CNS may occur due to the disruption of the BBB (65). Furthermore, the positive correlation between Gal-9 with ARTN concentrations, previously associated with cognitive failure and pain symptoms (29), adds another layer to the intricate network of molecular interactions in LC. GFRα3, the major ARTN receptor, is highly expressed in sensory and sympathetic ganglia of the peripheral nervous system (46) but not in immune cells (our unpublished observations). Similarly, ARTN is observed in human tissues (e.g. kidney and lung), Schwann cells, and upregulated after nerve injury, which implies glia are the main source of this neurotrophic factor (46). Therefore, it is so complex to delineate the role of ARTN in this context. On one hand, we have shown that the plasma ARTN concentrations are positively associated with cognitive impairments and pain scores in LC patients (29). On the other hand, ARTN might function as a compensatory mechanism to repair neural damage in LC patients by ongoing inflammation. Alternatively, we have reported that the expanded CD71+ erythroid cells (CECs) (66, 67) in the peripheral blood of LC patients by secretion of ARTN may play a role in this scenario (29), as reported by CECs in animal cancer models (68). However, it is unclear whether Gal-9 directly or indirectly influences ARTN expression in tissues. Therefore, the correlation of Gal-9 with ARTN merits further investigations.

Despite a previous report that Gal-9 levels were elevated in the plasma and CSF of PLWH (45), we found a significant reduction in the plasma ARTN concentrations in PLWH compared to HCs and LC patients. This observation indicates that Gal-9 does not have ever an absolute control over ARTN in PLWH. The expansion of CECs in the peripheral blood of PLWH (69) alongside the reduction in ARTN, contradicts their role as a major source of this neurotrophic factor. This discrepancy between SARS-CoV-2 and HIV might be explained by differential viral pathogenicity, chronicity, or other unknown mechanisms. Notably, recent studies have indicated the persistent SARS-CoV-2 RNA in LC patients up to several months post the onset of acute disease (12, 70). Nevertheless, our studies were performed 12 months after the acute SARS-CoV-2 infection, and at this stage, we are unaware of viral persistence in our LC cohort. Alternatively, the elevation of ARTN levels likely reflects a response to chronic inflammation, immune dysregulation, and nerve damage, as the body attempts to repair neuronal damage in LC patients. In contrast, reduced ARTN levels in HIV are likely due to the overall impaired neuroimmune responses and potential direct effects of the virus or inflammation on ARTN production. Therefore, further studies are required to determine other ARTN sources, such as endothelial cells and peripheral neurons, in LC patients with ME/CFS (64).

**Figure 4:**
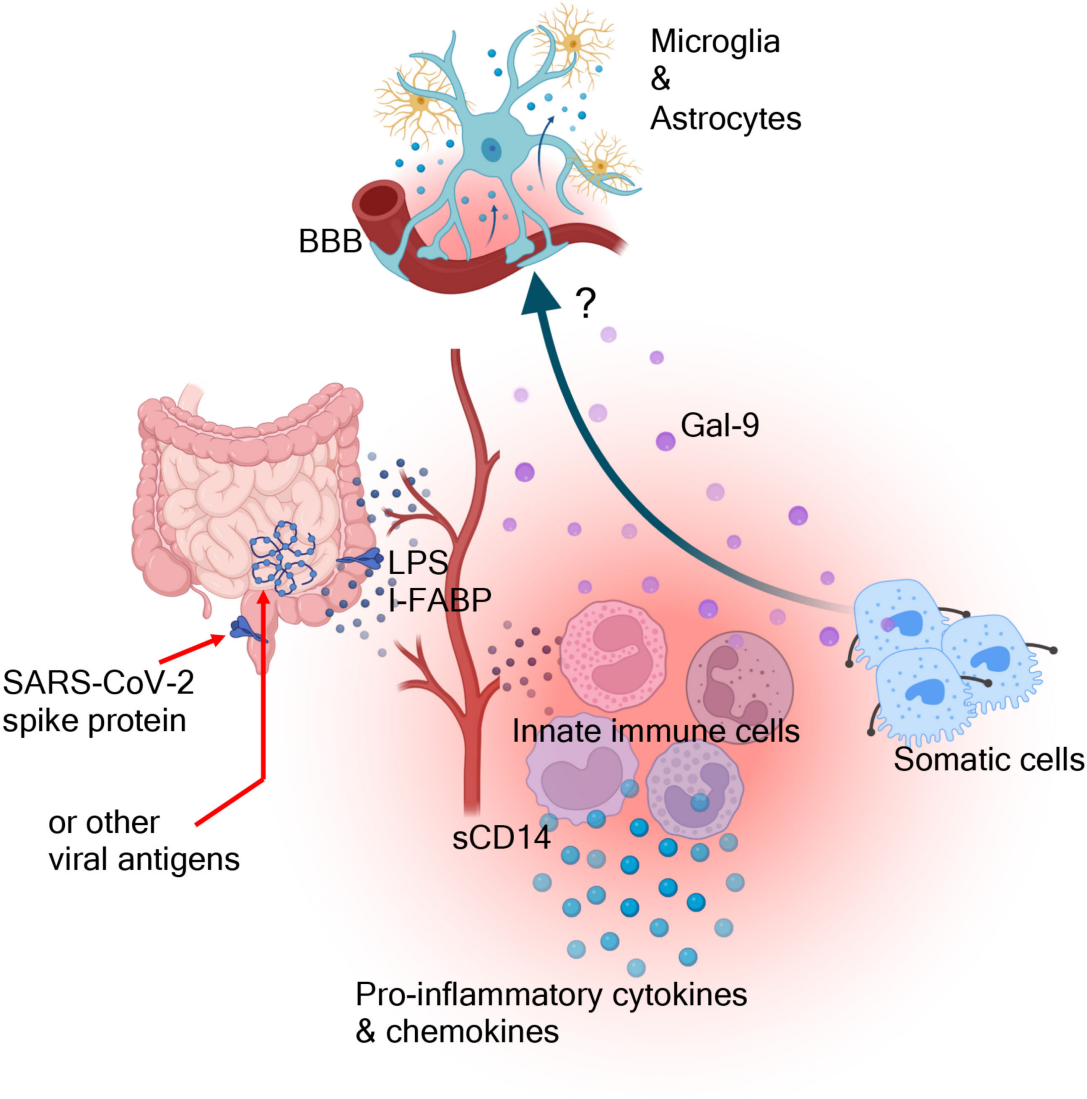
The proposed graphic summary. The gastrointestinal involvement during acute SARS-CoV-2 infection and the possible persistence of viral antigen/replication beyond the acute phase result in compromised gut barrier integrity. This, subsequently, leads to the translocation of microbial by-products (e.g. LPS) into the blood circulation. This leads to the activation of innate immune cells and the release of variety of pro-inflammatory cytokines and chemokines. This inflammatory cascade may result in immune/non-immune cell apoptosis and the release of damage-associated molecular patterns (e.g. Gal-9). Gal-9 may influence the activation/deactivation of different immune cells and ultimately may directly/indirectly influence the effector functions of microglia and astrocytes.

We propose that compromised gut barrier integrity occurs in a subset of SAR-CoV-2 infected individuals at the onset of acute disease or alternatively due to persistent viral replication and/or the presence of viral antigens in the gut of LC patients (**Figure 4**). This leads to microbial translocation to the periphery, which subsequently results in the activation of innate immune cells. This is supported by the elevation of LPS-BP, I-FABP, and sCD14 in the plasma of LC patients. Activated innate immune cells secrete pro-inflammatory cytokines and chemokines, contributing to systemic inflammation in LC patients (**Figure 4**). Additionally, Gal-9, released by immune and non-immune cells due to its immunomodulatory properties, exacerbate immune dysregulation. Finally, elevated plasma Gal-9 and pro-inflammatory biomarkers may directly or indirectly influence the CNS due to BBD disruption in LC patients (**Figure 4**). In conclusion, our findings highlight the potential of plasma Gal-9 and/or ARTN as sensitive biomarkers for identifying and stratifying LC individuals with ME/CFS. The correlations with inflammatory markers, immune activation, and cognitive impairment underscore the multifaceted nature of Gal-9 in LC, providing valuable insights for future research and potential therapeutic interventions. However, we are aware of several study limitations. This is a descriptive study, and further investigations are needed to understand how Gal-9 and/or ARTN affect cognitive impairments. Additionally, similar studies in larger cohorts, particularly, longitudinal studies are needed to confirm our findings. The heterogeneity of LC patients in terms of clinical presentations should be considered in future studies, given that the majority of our LC patients had a mild acute infection. Considering the differential immunological effects of SARS-CoV-2 strains, such as the Wuhan strain compared to the Delta and Omicron variants (44), it is important to examine how these variants might differentially impact LC syndrome. Our discovery cohort consisted of individuals infected with the Wuhan strain, while our validating cohort primarily included subjects infected with the Delta and Omicron variants. Therefore, future studies should account for the potential differential effects of SARS-CoV-2 variants of concerns. Finally, given the chronic systemic immune dysregulation/activation and potential cross-talk between HIV and SARS-CoV-2 (71), it would be informative to determine whether PLWH are more prone to have worst outcome when they become infected by SARS-CoV-2 virus or to developing LC syndrome.

## Data Availability

The original contributions presented in the study are included in the article/supplementary material. Further inquiries can be directed to the corresponding author.

## References

[1] Davis HE McCorkell L Vogel JM Topol EJ. Long COVID: major findings, mechanisms and recommendations. Nat Rev Microbiol. (2023) 21:133–46. doi: 10.1038/s41579-022-00846-2 PMC9839201 36639608

[2] Glynne P Tahmasebi N Gant V Gupta R. Long COVID following mild SARS-CoV-2 infection: characteristic T cell alterations and response to antihistamines. J Investig Med. (2022) 70:61–7. doi: 10.1136/jim-2021-002051 PMC8494538 34611034

[3] Gorna R MacDermott N Rayner C O'Hara M Evans S Agyen L. Long COVID guidelines need to reflect lived experience. Lancet. (2021) 397:455–7. doi: 10.1016/S0140-6736(20)32705-7 PMC7755576 33357467

[4] Saito S Shahbaz S Luo X Osman M Redmond D Cohen Tervaert JW. Metabolomic and immune alterations in long COVID patients with chronic fatigue syndrome. Front Immunol. (2024) 15:1341843. doi: 10.3389/fimmu.2024.1341843 38304426 PMC10830702

[5] Bai F Tomasoni D Falcinella C Barbanotti D Castoldi R Mulè G. Female gender is associated with long COVID syndrome: a prospective cohort study. Clin Microbiol Infect. (2022) 28:611.e9–611.e16. doi: 10.1016/j.cmi.2021.11.002 PMC8575536 34763058

[6] Cairns R Hotopf M. A systematic review describing the prognosis of chronic fatigue syndrome. Occup Med (Lond). (2005) 55:20–31. doi: 10.1093/occmed/kqi013 15699087

[7] Haffke M Freitag H Rudolf G Seifert M Doehner W Scherbakov N. Endothelial dysfunction and altered endothelial biomarkers in patients with post-COVID-19 syndrome and chronic fatigue syndrome (ME/CFS). J Trans Med. (2022) 20:138. doi: 10.1186/s12967-022-03346-2 PMC8938726 35317812

[8] Klein J Wood J Jaycox JR Dhodapkar RM Lu PW Gehlhausen JR. Distinguishing features of long COVID identified through immune profiling. Nature. (2023) 623:139–48. doi: 10.1038/s41586-023-06651-y PMC10620090 37748514

[9] Sapkota HR Nune A. Long COVID from rheumatology perspective - a narrative review. Clin Rheumatol. (2022) 41:337–48. doi: 10.1007/s10067-021-06001-1 PMC8629735 34845562

[10] Vollbracht C Kraft K. Oxidative stress and hyper-inflammation as major drivers of severe COVID-19 and long COVID: implications for the benefit of high-dose intravenous vitamin C. Front Pharmacol. (2022) 13:899198. doi: 10.3389/fphar.2022.899198 35571085 PMC9100929

[11] Yin K Peluso MJ Luo X Thomas R Shin MG Neidleman J. Long COVID manifests with T cell dysregulation, inflammation and an uncoordinated adaptive immune response to SARS-CoV-2. Nat Immunol. (2024) 25:218–25. doi: 10.1038/s41590-023-01724-6 PMC10834368 38212464

[12] Zuo W He D Liang C Du S Hua Z Nie Q. The persistence of SARS-CoV-2 in tissues and its association with long COVID symptoms: a cross-sectional cohort study in China. Lancet Infect Dis. (2024) 24(8):845–55. doi: 10.1016/S1473-3099(24)00171-3 38663423

[13] Dani M Dirksen A Taraborrelli P Torocastro M Panagopoulos D Sutton R. Autonomic dysfunction in 'long COVID': rationale, physiology and management strategies. Clin Med (Lond). (2021) 21:e63–e7. doi: 10.7861/clinmed.2020-0896 PMC7850225 33243837

[14] Greene C Connolly R Brennan D Laffan A O'Keeffe E Zaporojan L. Blood-brain barrier disruption and sustained systemic inflammation in individuals with long COVID-associated cognitive impairment. Nat Neurosci. (2024) 27:421–32. doi: 10.1038/s41593-024-01576-9 PMC10917679 38388736

[15] Aschman T Mothes R Heppner FL Radbruch H. What SARS-CoV-2 does to our brains. Immunity. (2022) 55:1159–72. doi: 10.1016/j.immuni.2022.06.013 PMC9212726 35777361

[16] Quan N Whiteside M Herkenham M. Time course and localization patterns of interleukin-1beta messenger RNA expression in brain and pituitary after peripheral administration of lipopolysaccharide. Neuroscience. (1998) 83:281–93. doi: 10.1016/s0306-4522(97)00350-3 9466417

[17] Sparkman NL Buchanan JB Heyen JR Chen J Beverly JL Johnson RW. Interleukin-6 facilitates lipopolysaccharide-induced disruption in working memory and expression of other proinflammatory cytokines in hippocampal neuronal cell layers. J Neurosci. (2006) 26:10709–16. doi: 10.1523/JNEUROSCI.3376-06.2006 PMC6674759 17050710

[18] van Dam AM Brouns M Louisse S Berkenbosch F. Appearance of interleukin-1 in macrophages and in ramified microglia in the brain of endotoxin-treated rats: a pathway for the induction of non-specific symptoms of sickness? Brain Res. (1992) 588:291–6. doi: 10.1016/0006-8993(92)91588-6 1393581

[19] Merani S Chen W Elahi S. The bitter side of sweet: the role of Galectin-9 in immunopathogenesis of viral infections. Rev Med virology. (2015) 25:175–86. doi: 10.1002/rmv.1832 25760439

[20] Dunsmore G Rosero EP Shahbaz S Santer DM Jovel J Lacy P. Neutrophils promote T-cell activation through the regulated release of CD44-bound Galectin-9 from the cell surface during HIV infection. PloS Biol. (2021) 19:e3001387. doi: 10.1371/journal.pbio.3001387 34411088 PMC8407585

[21] Elahi S Niki T Hirashima M Horton H. Galectin-9 binding to Tim-3 renders activated human CD4+ T cells less susceptible to HIV-1 infection. Blood. (2012) 119:4192–204. doi: 10.1182/blood-2011-11-389585 PMC3359739 22438246

[22] Okoye I Xu L Motamedi M Parashar P Walker JW Elahi S. Galectin-9 expression defines exhausted T cells and impaired cytotoxic NK cells in patients with virus-associated solid tumors. J Immunotherapy Cancer. (2020) 8:e001849. doi: 10.1136/jitc-2020-001849 PMC7735134 33310773

[23] Rahmati ABS Elahi S. Galectin-9 promotes natural killer cells activity via interaction with CD44. Front Immunol. (2023) 14:1131379. doi: 10.3389/fimmu.2023.1131379 37006235 PMC10060867

[24] Shahbaz S Dunsmore G Koleva P Xu L Houston S Elahi S. Galectin-9 and VISTA expression define terminally exhausted T cells in HIV-1 infection. J Immunol. (2020) 204:2474–91. doi: 10.4049/jimmunol.1901481 32205423

[25] Bozorgmehr N Hnatiuk M Peters AC Elahi S. Depletion of polyfunctional CD26(high)CD8(+) T cells repertoire in chronic lymphocytic leukemia. Exp Hematol Oncol. (2023) 12:13. doi: 10.1186/s40164-023-00375-5 36707896 PMC9881277

[26] Lee K Elahi S Mashhouri S Ye C. Gout presenting as a chronic inflammatory arthritis from immune checkpoint inhibitors: case series. Rheumatology. (2021) 60:E441–E3. doi: 10.1093/rheumatology/keab608 34320630

[27] Perez Rosero E Heron S Jovel J O'Neil CR Turvey SL Parashar P. Differential signature of the microbiome and neutrophils in the oral cavity of HIV-infected individuals. Front Immunol. (2021) 12:780910. doi: 10.3389/fimmu.2021.780910 34858437 PMC8630784

[28] Bozorgmehr N Mashhouri S Perez Rosero E Xu L Shahbaz S Sligl W. Galectin-9, a player in cytokine release syndrome and a surrogate diagnostic biomarker in SARS-CoV-2 infection. mBio. (2021) 12:e00384–21. doi: 10.1128/mBio.00384-21 PMC8262904 33947753

[29] Saito S Shahbaz S Osman M Redmond D Bozorgmehr N Rosychuk RJ. Diverse immunological dysregulation, chronic inflammation, and impaired erythropoiesis in long COVID patients with chronic fatigue syndrome. J Autoimmun. (2024) 147:103267. doi: 10.1016/j.jaut.2024.103267 38797051

[30] Bogariu AM Dumitrascu DL. Digestive involvement in the Long-COVID syndrome. Med Pharm Rep. (2022) 95:5–10. doi: 10.15386/mpr-2340 35720240 PMC9177081

[31] Zollner A Koch R Jukic A Pfister A Meyer M Rössler A. Postacute COVID-19 is characterized by gut viral antigen persistence in inflammatory bowel diseases. Gastroenterology. (2022) 163:495–506.e8. doi: 10.1053/j.gastro.2022.04.037 35508284 PMC9057012

[32] Brenchley JM. Mucosal immunity in human and simian immunodeficiency lentivirus infections. Mucosal Immunol. (2013) 6:657–65. doi: 10.1038/mi.2013.15 PMC4154146 23549448

[33] Shieh A Epeldegui M Karlamangla AS Greendale GA. Gut permeability, inflammation, and bone density across the menopause transition. JCI Insight. (2020) 5:e134092. doi: 10.1172/jci.insight.134092 31830000 PMC7098720

[34] Premeaux TA Yeung ST Pillai SK Ndhlovu LC. Elevated Galectin-9 across the human brain correlates with HIV neuropathology and detrimental cognitive states. J Neurovirol. (2023) 29:337–45. doi: 10.1007/s13365-023-01149-9 37233903

[35] Minnema L Wheeler J Enomoto M Pitake S Mishra SK Lascelles BDX. Correlation of artemin and GFRalpha3 with osteoarthritis pain: early evidence from naturally occurring osteoarthritis-associated chronic pain in dogs. Front Neurosci. (2020) 14:77. doi: 10.3389/fnins.2020.00077 32116521 PMC7031206

[36] Gardell LR Wang R Ehrenfels C Ossipov MH Rossomando AJ Miller S. Multiple actions of systemic artemin in experimental neuropathy. Nat Med. (2003) 9:1383–9. doi: 10.1038/nm944 14528299

[37] Moar P Linn K Premeaux TA Bowler S Sardarni UK Gopalan BP. Plasma Galectin-9 relates to cognitive performance and inflammation among adolescents with vertically acquired HIV. Aids. (2024) 38:1460–7. doi: 10.1097/QAD.0000000000003907 PMC11239096 38608008

[38] Elahi S Weiss RH Merani S. Atorvastatin restricts HIV replication in CD4+ T cells by upregulation of p21. Aids. (2016) 30:171–83. doi: 10.1097/QAD.0000000000000917 26645604

[39] Elahi S. Galectin-9, a lingering shadow in HIV's fight: the unseen battle of adolescents with perinatally-acquired HIV. Aids. (2024) 38:1589–91. doi: 10.1097/QAD.0000000000003941 38990316

[40] Jason LA Sunnquist M Brown A Evans M Vernon SD Furst J. Examining case definition criteria for chronic fatigue syndrome and myalgic encephalomyelitis. Fatigue. (2014) 2:40–56. doi: 10.1080/21641846.2013.862993 24511456 PMC3912876

[41] Lim EJ Ahn YC Jang ES Lee SW Lee SH Son CG. Systematic review and meta-analysis of the prevalence of chronic fatigue syndrome/myalgic encephalomyelitis (CFS/ME). J Trans Med. (2020) 18:100. doi: 10.1186/s12967-020-02269-0 PMC7038594 32093722

[42] Shahbaz S Okoye I Blevins G Elahi S. Elevated ATP via enhanced miRNA-30b, 30c, and 30e downregulates the expression of CD73 in CD8+ T cells of HIV-infected individuals. PloS Pathog. (2022) 18:e1010378. doi: 10.1371/journal.ppat.1010378 35325005 PMC8947394

[43] Bozorgmehr N Okoye I Oyegbami O Xu L Fontaine A Cox-Kennett N. Expanded antigen-experienced CD160(+)CD8(+)effector T cells exhibit impaired effector functions in chronic lymphocytic leukemia. J Immunother Cancer. (2021) 9(4):e002189. doi: 10.1136/jitc-2020-002189 33931471 PMC8098955

[44] Shahbaz S Bozorgmehr N Lu J Osman M Sligl W Tyrrell DL. Analysis of SARS-CoV-2 isolates, namely the Wuhan strain, Delta variant, and Omicron variant, identifies differential immune profiles. Microbiol Spectr. (2023) 11:e0125623. doi: 10.1128/spectrum.01256-23 37676005 PMC10581158

[45] Premeaux TA D'Antoni ML Abdel-Mohsen M Pillai SK Kallianpur KJ Nakamoto BK. Elevated cerebrospinal fluid Galectin-9 is associated with central nervous system immune activation and poor cognitive performance in older HIV-infected individuals. J Neurovirology. (2019) 25:150–61. doi: 10.1007/s13365-018-0696-3 PMC6506351 30478799

[46] Baloh RH Tansey MG Lampe PA Fahrner TJ Enomoto H Simburger KS. Artemin, a novel member of the GDNF ligand family, supports peripheral and central neurons and signals through the GFRα3-RET receptor complex. Neuron. (1998) 21:1291–302. doi: 10.1016/s0896-6273(00)80649-2 9883723

[47] Katoh S Ikeda M Shimizu H Fukushima K Oka M. Induction of galectin-9 production by viral infection in the lung. Eur Respir J. (2015) 46:OA1780. doi: 10.1183/13993003.congress-2015.OA1780

[48] Mengshol JA Golden-Mason L Arikawa T Smith M Niki T McWilliams R. A crucial role for Kupffer cell-derived galectin-9 in regulation of T cell immunity in hepatitis C infection. PloS One. (2010) 5:e9504. doi: 10.1371/journal.pone.0009504 20209097 PMC2831996

[49] Matsuoka N Kozuru H Koga T Abiru S Yamasaki K Komori A. Galectin-9 in autoimmune hepatitis Correlation between serum levels of galectin-9 and M2BPGi in patients with autoimmune hepatitis. Medicine. (2019) 98:e16924. doi: 10.1097/MD.0000000000016924 31464928 PMC6736219

[50] Elahi S Dinges WL Lejarcegui N Laing KJ Collier AC Koelle DM. Protective HIV-specific CD8+ T cells evade Treg cell suppression. Nat Med. (2011) 17:989–95. doi: 10.1038/nm.2422 PMC3324980 21765403

[51] Yang R Sun L Li C-F Wang Y-H Yao J Li H. Galectin-9 interacts with PD-1 and TIM-3 to regulate T cell death and is a target for cancer immunotherapy. Nat Commun. (2021) 12:832. doi: 10.1038/s41467-021-21099-2 33547304 PMC7864927

[52] Chen HY Wu YF Chou FC Wu YH Yeh LT Lin KI. Intracellular galectin-9 enhances proximal TCR signaling and potentiates autoimmune diseases. J Immunol. (2020) 204:1158–72. doi: 10.4049/jimmunol.1901114 31969388

[53] Motamedi M Shahbaz S Fu L Dunsmore G Xu L Harrington R. Galectin-9 expression defines a subpopulation of NK cells with impaired cytotoxic effector molecules but enhanced IFN-gamma production, dichotomous to TIGIT, in HIV-1 infection. Immunohorizons. (2019) 3:531–46. doi: 10.4049/immunohorizons.1900087 31732662

[54] Martin TR Wurfel MM Zanoni I Ulevitch R. Targeting innate immunity by blocking CD14: Novel approach to control inflammation and organ dysfunction in COVID-19 illness. Ebiomedicine. (2020) 57:102836. doi: 10.1016/j.ebiom.2020.102836 32574958 PMC7305752

[55] Cao VT Carter MC Brenchley JM Bolan H Scott LM Bai Y. sCD14 and intestinal fatty acid binding protein are elevated in the serum of patients with idiopathic anaphylaxis. J Allergy Clin Immunol Pract. (2023) 11:2080–6.e5. doi: 10.1016/j.jaip.2023.03.037 36997122 PMC10411508

[56] Vassallo M Mercié P Cottalorda J Ticchioni M Dellamonica P. The role of lipopolysaccharide as a marker of immune activation in HIV-1 infected patients: a systematic literature review. Virol J. (2012) 9:174. doi: 10.1186/1743-422X-9-174 22925532 PMC3495848

[57] Natarajan A Zlitni S Brooks EF Vance SE Dahlen A Hedlin H. Gastrointestinal symptoms and fecal shedding of SARS-CoV-2 RNA suggest prolonged gastrointestinal infection. Med. (2022) 3:371–87.e9. doi: 10.1016/j.medj.2022.04.001 35434682 PMC9005383

[58] Icard P Lincet H. A global view of the biochemical pathways involved in the regulation of the metabolism of cancer cells. Biochim Biophys Acta. (2012) 1826:423–33. doi: 10.1016/j.bbcan.2012.07.001 22841746

[59] Saito S Bozorgmehr N Sligl W Osman M Elahi S. The role of coinhibitory receptors in B cell dysregulation in SARS-CoV-2-infected individuals with severe disease. J Immunol. (2024) 212:1540–52. doi: 10.4049/jimmunol.2300783 PMC11075007 38517295

[60] Thomas T Stefanoni D Reisz JA Nemkov T Bertolone L Francis RO. COVID-19 infection alters kynurenine and fatty acid metabolism, correlating with IL-6 levels and renal status. JCI Insight. (2020) 5:e140327. doi: 10.1172/jci.insight.140327 32559180 PMC7453907

[61] Du L Bouzidi MS Gala A Deiter F Billaud JN Yeung ST. Human galectin-9 potently enhances SARS-CoV-2 replication and inflammation in airway epithelial cells. J Mol Cell Biol. (2023) 15(4):mjad030. doi: 10.1093/jmcb/mjad030 37127426 PMC10668544

[62] Bi SG Hong PW Lee B Baum LG. Galectin-9 binding to cell surface protein disulfide isomerase regulates the redox environment to enhance T-cell migration and HIV entry. Proc Natl Acad Sci United States America. (2011) 108:10650–5. doi: 10.1073/pnas.1017954108 PMC3127870 21670307

[63] Steelman AJ Li J. Astrocyte galectin-9 potentiates microglial TNF secretion. J Neuroinflammation. (2014) 11:144. doi: 10.1186/s12974-014-0144-0 25158758 PMC4158089

[64] Zhu S Li Y Bennett S Chen J Weng IZ Huang L. The role of glial cell line-derived neurotrophic factor family member artemin in neurological disorders and cancers. Cell Prolif. (2020) 53:e12860. doi: 10.1111/cpr.12860 32573073 PMC7377943

[65] Bonetto V Pasetto L Lisi I Carbonara M Zangari R Ferrari E. Markers of blood-brain barrier disruption increase early and persistently in COVID-19 patients with neurological manifestations. Front Immunol. (2022) 13:1070379. doi: 10.3389/fimmu.2022.1070379 36591311 PMC9798841

[66] Elahi S Vega-Lopez MA Herman-Miguel V Ramirez-Estudillo C Mancilla-Ramirez J Motyka B. CD71(+) erythroid cells in human neonates exhibit immunosuppressive properties and compromise immune response against systemic infection in neonatal mice. Front Immunol. (2020) 11:597433. doi: 10.3389/fimmu.2020.597433 33329589 PMC7732591

[67] Shahbaz S Xu L Osman M Sligl W Shields J Joyce M. Erythroid precursors and progenitors suppress adaptive immunity and get invaded by SARS-CoV-2. Stem Cell Rep. (2021) 16:1165–81. doi: 10.1016/j.stemcr.2021.04.001 PMC8111797 33979601

[68] Bozorgmehr N Okoye I Mashhouri S Lu J Koleva P Walker J. CD71(+) erythroid cells suppress T-cell effector functions and predict immunotherapy outcomes in patients with virus-associated solid tumors. J Immunother Cancer. (2023) 11:e006595. doi: 10.1136/jitc-2022-006595 37236637 PMC10230995

[69] Namdar A Koleva P Shahbaz S Strom S Gerdts V Elahi S. CD71+ erythroid suppressor cells impair adaptive immunity against Bordetella pertussis. Sci Rep. (2017) 7:7728. doi: 10.1038/s41598-017-07938-7 28798335 PMC5552872

[70] Menezes SM Jamoulle M Carletto MP Moens L Meyts I Maes P. Blood transcriptomic analyses reveal persistent SARS-CoV-2 RNA and candidate biomarkers in post-COVID-19 condition. Lancet Microbe. (2024) 5(8):100849. doi: 10.1016/S2666-5247(24)00055-7 38677304

[71] Shahbaz S Sligl W Osman M Elahi S. Immunological responses in SARS-CoV-2 and HIV co-infection versus SARS-CoV-2 mono-infection: case report of the interplay between SARS-CoV-2 and HIV. Allergy Asthma Cl Im. (2023) 19(1):91. doi: 10.1186/s13223-023-00846-8 PMC10583436 37848967

